# A Quantitative Framework for Defining the End of an Infectious Disease Outbreak: Application to Ebola Virus Disease

**DOI:** 10.1093/aje/kwaa212

**Published:** 2020-10-14

**Authors:** Bimandra A Djaafara, Natsuko Imai, Esther Hamblion, Benido Impouma, Christl A Donnelly, Anne Cori

**Keywords:** basic reproduction number, disease outbreaks, Ebola virus disease, end-of-outbreak declaration, epidemics

## Abstract

The end-of-outbreak declaration is an important step in controlling infectious disease outbreaks. Objective estimation of the confidence level that an outbreak is over is important to reduce the risk of postdeclaration flare-ups. We developed a simulation-based model with which to quantify that confidence and tested it on simulated Ebola virus disease data. We found that these confidence estimates were most sensitive to the instantaneous reproduction number, the reporting rate, and the time between the symptom onset and death or recovery of the last detected case. For Ebola virus disease, our results suggested that the current World Health Organization criterion of 42 days since the recovery or death of the last detected case is too short and too sensitive to underreporting. Therefore, we suggest a shift to a preliminary end-of-outbreak declaration after 63 days from the symptom onset day of the last detected case. This preliminary declaration should still be followed by 90 days of enhanced surveillance to capture potential flare-ups of cases, after which the official end of the outbreak can be declared. This sequence corresponds to more than 95% confidence that an outbreak is over in most of the scenarios examined. Our framework is generic and therefore could be adapted to estimate end-of-outbreak confidence for other infectious diseases.

## Abbreviations

EVD: Ebola virus disease
WHO: World Health Organization

Declaring the end of an outbreak is a critical programmatic step in outbreak response. Any infectious disease outbreak can be devastating for affected populations and areas. The outbreak status of a region or a country can influence other vital sectors, such as the social, economic, political, and security sectors (1–3). In defining the end of an outbreak, it is crucial to consider the risk of cases’ arising in the future using objective quantitative methods. A well-timed end-of-outbreak declaration is essential. It allows affected countries to address problems in other sectors, reallocate health-care resources to cover other important public health issues, and start postoutbreak recovery efforts.

Various end-of-outbreak definitions or criteria have been used in different outbreaks, depending on the type of disease and the institutions that are providing technical guidance or are in charge of controlling the outbreak. We retrieved examples of end-of-outbreak criteria for various pathogens and from various institutions from the literature (4–17) (Table 1). Most of these criteria were obtained from World Health Organization (WHO) guidelines or decided by a government’s Ministry of Health or public health authorities. Based on the most commonly used criterion, the end of an outbreak can be declared when a period of twice the longest incubation period without observing any new cases since the last possible transmission event has passed (4–12). In the context of an Ebola virus disease (EVD) outbreak, the end of an outbreak of EVD is declared following 42 consecutive days (twice the longest incubation period) of no cases’ being recorded since the outcome of the last detected case. The outcome is defined as the second polymerase chain reaction–negative test of blood samples or a safe burial if the person died (8).

**Table 1:** End-of-Outbreak Criteria Used in Past Infectious Disease Outbreaks

**First Author, Year** **(Reference No.)**	**Disease**	**Outbreak Location**	**Outbreak Year(s)**	**End-of-Outbreak Criterion**
Ministry of Health, Republic of Uganda, 2017 (4)	Marburg virus	Uganda	2017	
Normile, 2015 (5); Yonhap News Agency, 2018 (6)	MERS	South Korea	2015 and 2018	Twice the longest incubation period of no cases since the death/recovery of the last confirmed case
Republika Online, 2018 (7)	Diphtheria	Indonesia	2017–2018	
WHO, 2015 (8, 9); WHO, 2016 (10); WHO, 2017 (11); WHO, 2018 (12)	EVD	West Africa	2013–2018	
WHO, 2017 (13)	Lassa fever	Benin	2016	Twice the longest incubation period of no cases since the reporting day of the last confirmed case
WHO, 2017 (14)	Yellow fever	DRC and Angola	2015–2017	No new cases reported for 6 months
WHO, 2018 (15)	Cholera	South Sudan	2017–2018	No new cases reported for 7 weeks
WHO, 2018 (16)	Listeriosis	South Africa	2017–2018	No cases due to the outbreak strain reported for 3 months and incidence rate in the past 2 months that has dropped to the preoutbreak level
WHO, 2017 (17)	Meningitis	Nigeria	2016–2017	Weekly number of reported cases below the “epidemic and alert threshold” for 8 weeks

Abbreviations: DRC, Democratic Republic of the Congo; EVD, Ebola virus disease; MERS, Middle East respiratory syndrome; WHO, World Health Organization.

The use of this criterion to declare the end of an outbreak, especially in the context of EVD, has been questioned. Firstly, this criterion ignores the possible recrudescence of EVD cases through less common transmission routes, such as sexual transmission, immunocompromised women, and migration (18–22). Recrudescence of EVD cases has been problematic in the field; for example, in 2015–2016, there were 3 end-of-outbreak declarations in Liberia before the outbreak was actually over (21). Secondly, the use of the maximum incubation period is challenging, since typically limited sample sizes are available for estimation of that duration, and it does not produce any probabilistic risk assessment (23). Lastly, it is important to consider unreported cases due to imperfect surveillance and asymptomatic cases. They could potentially act as invisible transmission sources during the outbreak and prolong the time to the end-of-outbreak declaration (23, 24).

Quantitative frameworks that account for these issues are therefore needed to objectively estimate the confidence (for example, probabilistically, 95% certain) that there will be no postdeclaration flare-ups of cases. However, to date, only a few studies have focused on developing quantitative methods with which to define end-of-outbreak criteria, mostly for directly transmitted or airborne pathogens. Nishiura et al. (24) developed a probabilistic method for calculating the probability of observing additional cases of Middle East respiratory syndrome in the future. The method accounts for the serial interval (the time between symptom onset in a case and the case’s infector) and the basic reproduction number (the average number of secondary infections generated by a single case in a completely susceptible population) of Middle East respiratory syndrome. Eichner and Dietz (25) used stochastic simulations to determine the length of the case-free period before declaring the extinction of poliovirus with a specified error probability. Thompson et al. (26) used stochastic susceptible-exposed-infectious-recovered model simulations to assess the influence of underreporting of EVD cases on the confidence of an EVD end-of-outbreak declaration.

However, these approaches only address some of the aforementioned issues and have other limitations, which we discuss below. Hence, further development of quantitative techniques for defining the end of an outbreak is urgently needed. In this study, we developed a simulation-based method for calculating the confidence that an outbreak is over after the outcome of the last detected case is known. We accounted for factors that influence the estimated confidence: the underlying reproduction number, the reporting rate, and the time between the symptom onset and the outcome (recovery or death) of the last detected case. For simplicity, we refer to the latter as the “onset-to-outcome delay phase,” where “onset” is the symptom onset day and “outcome” is recovery from the disease or the death of the case. We tested our method on several EVD outbreak scenarios. Finally, we used the simulation results to propose a new quantitative criterion for defining the end of an outbreak for EVD.

## METHODS

**Figure 1:**
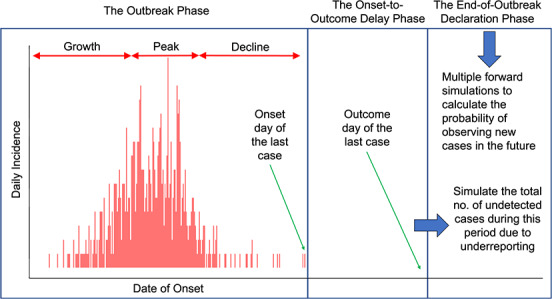
Quantitative framework for defining the end of an infectious disease outbreak. The 3 main phases of the framework are 1) the outbreak phase ($t=1,2,\ldots, T$; $T$ = the observed duration of the outbreak so far); 2) the onset-to-outcome delay phase ($d=1,2,\ldots, D$; $D$ = the length of the onset-to-outcome delay phase of the last detected case); and 3) the end-of-outbreak declaration phase ($z=1,2,\ldots, S;S=$ the number of days needed to reach the desired probability threshold of cases’ arising in the future).

We developed a quantitative framework to determine the timing of an end-of-outbreak declaration, which divides the outbreak into 3 phases: 1) the outbreak phase; 2) the onset-to-outcome delay phase; and 3) the end-of-outbreak declaration phase (Figure 1). The outbreak phase encompasses the outbreak trajectory, which includes all cases detected up to the time that the end-of-outbreak analysis is about to be conducted. The onset-to-outcome delay phase is the period between the symptom onset of the last detected case and the outcome (recovery or death) of that case. In this phase, there is a risk of undetected cases’ sustaining transmission beyond the last detected case due to underreporting. Accounting for these potential “invisible” sources of transmission is important for determining the end of the outbreak with confidence, irrespective of potential underreporting. The end-of-outbreak declaration phase starts the day after the outcome of the last detected case, where the confidence that the outbreak is over is calculated for each day. We used 95% confidence as a threshold for declaring that an outbreak was over. The Web Appendix (see Web Figure 1, available at https://doi.org/10.1093/aje/kwaa212) shows the simulation process.

### The outbreak phase

We simulated outbreak data using the *project* function from the *projections* package (27) in R software (R Foundation for Statistical Computing, Vienna, Austria) (28), with epidemic parameters taken from the 2013–2016 EVD epidemic in West Africa (29). We assumed either Poisson or negative binomial offspring distributions (“offspring” corresponds to new infections caused by an infected individual) to allow for overdispersion (or superspreading, whereby a few cases are responsible for a large proportion of infections due to individual-level variation in transmission). The statistical formulation of the outbreak simulations is as follows: ${I}_t\sim \mathrm{Poisson}({R}_t{\lambda}_t)$ or ${I}_t\sim \mathrm{NegBin}({R}_t{\lambda}_t,k)$, where ${I}_t$ is the number of new cases arising (based on symptom onset) at time *t*, ${R}_t$ is the instantaneous reproduction number at time *t* (i.e., the average number of secondary infections generated by a case arising at time *t*, if conditions remain the same), *k* is the overdispersion parameter for the negative binomial distribution, and ${\lambda}_t$ is the total infectiousness in the population at time *t*. ${\lambda}_t$ is described by $\omega$ and ${I}_t$ as

(1) \begin{equation*} {\lambda}_t=\sum \limits_{s=1}^t{I}_{t-s}{\omega}_s, \end{equation*}

where ${\omega}_s$ is the typical infectivity profile (approximated by the serial interval distribution) of a case at time $s$ after symptom onset.

The case incidence for the first 30 days of the simulated outbreak were taken from the *ebola_sim* line list data from the *outbreaks* R package (30). These data are simulated EVD outbreak data with key properties that match those of the 2013–2016 EVD epidemic in West Africa. After the first 30 days, stochastic simulations were carried out using the model described above. The serial interval was assumed to be gamma-distributed with a mean of 15.3 days and a standard deviation of 9.3 days (29). For the negative binomial simulations, the overdispersion parameter *k* was set to values in the range 0.03–0.52, consistent with estimates from the West African EVD epidemic (31, 32). We assumed that ${R}_t$ varied over time, with the outbreak divided into 3 periods: growth, peak, and decline. The “growth” period included the initial 30 days, with an ${R}_t$ of 1.7 (within the range of Van Kerkhove et al.’s (29) estimates) and was assumed to last for a total of 90 days. During the “peak” period, assumed to last for 40 days, we used ${R}_t$ = 1.0. The “decline” period, with ${R}_t$ values in the range of 0.3–0.9, lasted until the simulated outbreak trajectory had no more cases. We simulated 100 stochastic outbreak trajectories for each scenario considered (see “Simulation scenarios” subsection below).

### The onset-to-outcome delay phase

It is important to account for the possible new infections caused by undetected cases during the period between the symptom onset and the outcome of the last detected case (the “onset-to-outcome delay phase”). We simulated the number of undetected cases in that period using a probabilistic method, under several underreporting assumptions. Using Bayes’ theorem, an inverse binomial problem was solved to calculate the probability distribution of the total number of cases arising during the onset-to-outcome delay phase, given the reporting rate and 0 cases detected during that period. The probability mass function of the binomial distribution is described as

(2) \begin{equation*} f\left(x|n,p\right)=\frac{n!}{\left(n-x\right)!x!}{p}^x{\left(1-p\right)}^{n-x}, \end{equation*}

where $n$ is the total number of cases (detected and undetected) during the onset-to-outcome delay phase, *p* is the reporting rate, and *x* is the number of cases detected during that period, which is 0 by definition. Bayes’ theorem was used to solve the inverse binomial problem of inferring *n*, given the value of *x* and *p*: $f(n|x,p)\propto f(x|n,p)f(n)$, with $f(n|x,p)$ as the posterior distribution of *n* given the value of *x* and *p*, $f(x|n,p)$ as the binomial likelihood, and $f(n)$ as the prior distribution for *n*.

The prior distribution $f(n)$ was generated by simulating outbreaks in various hypothetical scenarios with different instantaneous reproduction numbers and reporting rates. For each simulation, 10,000 forward trajectories of 21 days starting a day after the onset day of the last case of simulated data sets were generated. Conservatively, 21 days was assumed as the maximum length of the onset-to-outcome delay. This assumed that the maximum length accounts for both the average onset-to-death delay and the average onset-to-recovery delay (upper bound of the onset-to-outcome delay). These delay durations during the West African EVD epidemic were estimated as 8.2 days and 15.1 days, respectively, with the majority of delays being less than 21 days (33).

The number of undetected cases during the onset-to-outcome delay phase was obtained by solving the inverse binomial problem described above. Those cases were allocated probabilistically to each day within this period using a multinomial distribution,

(3) \begin{align*} &f\left({u}_1,\ldots, {u}_D|n,{p}_1,\ldots, {p}_D\right)\nonumber\\&\quad=\frac{y!}{u_1!{u}_2!\ldots{u}_D!}{p}_1^{u_1}{p}_2^{u_2}\ldots{p}_D^{u_D}, \end{align*}

where *y* is the total number of undetected cases obtained by the inverse binomial problem, ${u}_1,\ldots, {u}_D$ are the number of undetected cases on day $d$ ($d=1,2,\ldots, D$) after the onset of the last detected case, and ${p}_1,\ldots, {p}_D$ are the probabilities of the undetected cases appearing on day *d* ($d=1,2,\cdots, D$). ${p}_1,\ldots, {p}_D$ were calculated by dividing the total infectiousness on each day by the sum of the daily total infectiousness during the whole period of *D* days:

(4) \begin{equation*} {p}_d=\frac{\lambda_d}{\sum_{s=1}^D{\lambda}_s},d=1,2,\ldots, D, \end{equation*}

where $\lambda$ is defined as in equation 1. For each simulated outbreak data set, we simulated the number of undetected cases 10 times. Random allocations were also simulated 10 times for each simulated number of undetected cases.

### The end-of-outbreak declaration phase

In the end-of-outbreak declaration phase, we performed forward projections of daily incidence of cases, irrespective of reporting status (${Y}_z$), for 300 days ($z=1,2,\ldots, 300$) following the outcome day of the last detected case. The projections are based on the combination of the outbreak trajectory and simulated undetected cases during the onset-to-outcome delay phase. We considered 2 transmission scenarios in the analysis. The first was the perfect-case-isolation scenario, where only the last detected case and subsequent undetected cases could contribute to onward transmissions. This scenario demonstrates the condition that exists if all previous cases have been perfectly isolated. The second scenario was the no-case-isolation scenario, where all previous cases (detected and undetected) and subsequent undetected cases can contribute to onward transmissions, reflecting no case isolation. The infectiousness of each case in these scenarios is assumed to follow the infectivity profile (${\omega}_s$) approximated by the serial interval distribution. Cases will be most infectious at time $s$ after the symptom onset, when ${\omega}_s$ is the largest (34). To calculate the end-of-outbreak confidence on day *z* after the death or recovery (“outcome”) of the last case, for each simulated outbreak data set, we made 10 forward projections for each combination of the simulated number of undetected cases and daily allocations of undetected cases.

For each projected trajectory, we calculated the total number of projected cases (irrespective of reporting status) from day *z* to 300 days after the outcome of the last case (${C}_z$) as

$$ {C}_z=\sum \limits_{s=z}^{300}{Y}_z. $$

The presence or absence of new cases on day $z$ after the outcome of the last case was then summarized as

$$ {X}_z=\left\{\begin{array}{@{}c} 1,\kern0.5em {C}_z>0\ \\ 0,\kern0.5em {C}_z=0.\end{array}\right. $$

The probability of cases’ arising in the future on day *z* after the outcome of the last case (${P}_z$) was then calculated as

$$ {P}_z=\frac{\sum_{i=1}^N{X}_{z,i}}{N}, $$

where *i* = 1, … *N* are the different trajectories simulated for a given scenario. We defined the confidence that an outbreak is over on day $z$ after the outcome of the last case as $1-{P}_z$.

### Simulation scenarios

We simulated several outbreak scenarios with different offspring distributions assuming either perfect case isolation or no case isolation. Table 2 shows the offspring distribution parameters in the “decline” period of the outbreak used for the simulations. For the primary analyses, we simulated the framework using the same ${R}_t$ value as the simulated outbreak data. We then calculated the end-of-outbreak confidence, $1-{P}_z$ ($z=1,2,\ldots, 300$), to define *Z* (the waiting time for end-of-outbreak declaration), where $(1-{P}_z)>95\%$.

**Table 2:** Offspring Distribution Parameters in the “Decline” Period of an Outbreak Used for End-of-Outbreak Simulations

**Offspring Probability** **Distribution**	***R***_***t***_ ^**a**^	***k*** ^**b**^	**Source for *k*** **(First Author, Year** **(Reference No.))**
Poisson	0.6		
Poisson	0.3		
Poisson	0.9		
Negative binomial	0.6	0.52 (low overdispersion)	IERT, 2016 (32)
Negative binomial	0.6	0.18 (medium overdispersion)	Althaus, 2015 (31)
Negative binomial	0.6	0.03 (high overdispersion)	IERT, 2016 (32)

Abbreviation: IERT, International Ebola Response Team.

^a^ Instantaneous reproduction number.

^b^ Overdispersion parameter for negative binomial distribution.

We conducted sensitivity analyses on the robustness of the framework to the misspecification of the value of ${R}_t$ in the “decline” period. We performed further simulations using underestimated (0.3) and overestimated (0.9) ${R}_t$ values for simulated outbreak data with true ${R}_t$ = 0.6 in the “decline” period. Web Table 1 shows the complete combination of all simulation scenarios explored.

## RESULTS

Using the quantitative simulation framework developed, we estimated the confidence that an outbreak was over on various numbers of days after the outcome of the last detected case. We considered different scenarios (summarized in Web Table 1) by varying the offspring distribution of the outbreak, the time between the symptom onset to recovery or death of the last detected case (the onset-to-outcome delay phase), the reporting rate, and the case isolation assumption (perfect case isolation or no case isolation). Figure 2 shows, for 6 different offspring distributions, the number of days taken (from the outcome of the last detected case) to reach 95% confidence that an outbreak was over.

**Figure 2 Continues:**
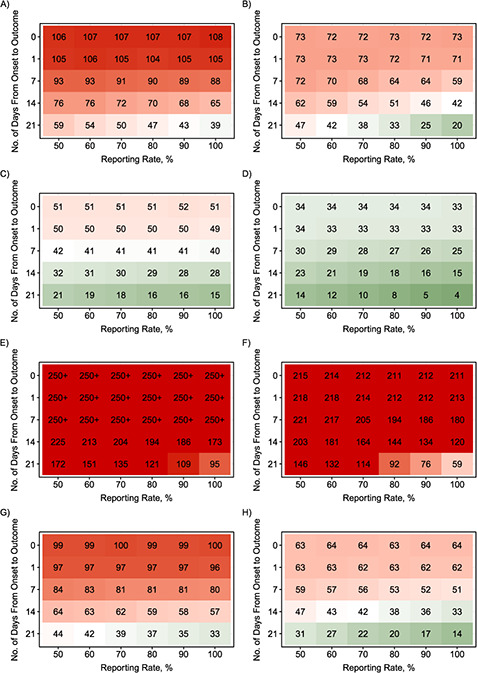


**Figure 2:**
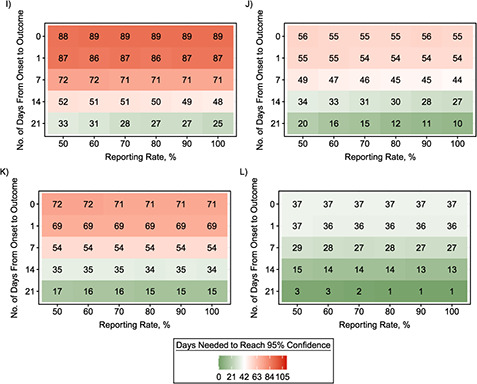
Amount of time (days from the outcome of the last detected case) needed until the calculated end-of-outbreak confidence reaches 95% as a function of the reporting rate and the length of the onset-to-outcome delay phase for combinations of transmission scenarios and various offspring distributions during the “decline” period. A) No case isolation with Poisson-based ${R}_t$ = 0.6; B) perfect case isolation with Poisson-based ${R}_t$ = 0.6; C) no case isolation with Poisson-based ${R}_t$ = 0.3; D) perfect case isolation with Poisson-based ${R}_t$ = 0.3; E) no case isolation with Poisson-based ${R}_t$ = 0.9; F) perfect case isolation with Poisson-based ${R}_t$ = 0.9; G) no case isolation with negative-binomial (NB)–based ${R}_t$ = 0.6 and $k=$ 0.52; H) perfect case isolation with NB-based ${R}_t$ = 0.6 and $k=$ 0.52; I) no case isolation with NB-based ${R}_t$ = 0.6 and $k=$ 0.18; J) perfect case isolation with NB-based ${R}_t$ = 0.6 and $k=$ 0.18; K) no case isolation with NB-based ${R}_t$ = 0.6 and $k=$ 0.03; L) perfect case isolation with NB-based ${R}_t$ = 0.6 and $k=$ 0.03. An onset-to-outcome delay of 0 days corresponds to counting days from the date of symptom onset of the last detected case. Red cells denote longer waiting times to reach 95% end-of-outbreak confidence, while green cells denote shorter waiting times. The current World Health Organization criterion is 42 days after the outcome of the last detected case.

**Figure 3 Continues:**
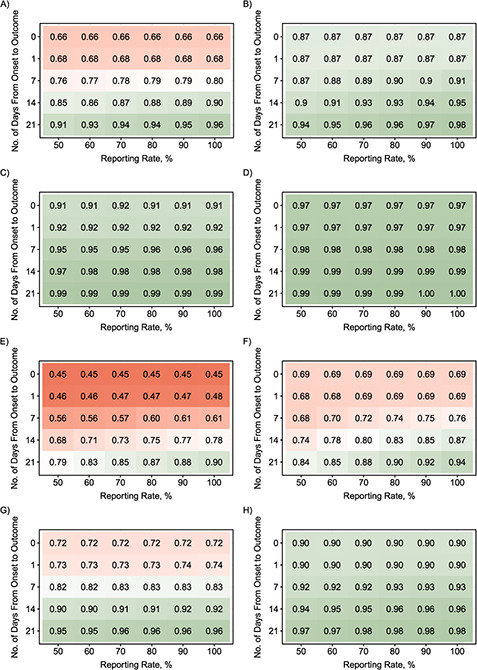


**Figure 3:**
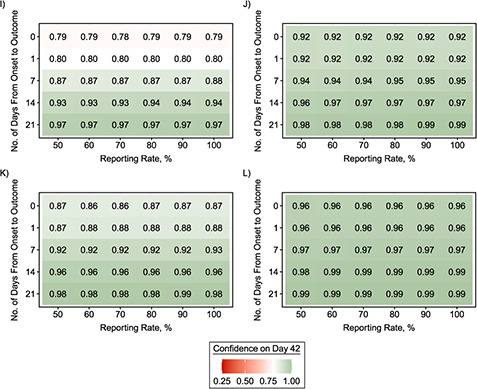
Level of confidence (shown as a proportion) that an outbreak is over following a 42-day period of no cases’ being detected after the outcome of the last detected case as a function of the reporting rate and the length of the onset-to-outcome delay phase for combinations of transmission scenarios and various offspring distributions during the “decline” period. A) No case isolation with Poisson-based ${R}_t$ = 0.6; B) perfect case isolation with Poisson-based ${R}_t$ = 0.6; C) no case isolation with Poisson-based ${R}_t$ = 0.3; D) perfect case isolation with Poisson-based ${R}_t$ = 0.3; E) no case isolation with Poisson-based ${R}_t$ = 0.9; F) perfect case isolation with Poisson-based ${R}_t$ = 0.9; G) no case isolation with negative-binomial (NB)–based ${R}_t$ = 0.6 and $k=$ 0.52; H) perfect case isolation with NB-based ${R}_t$ = 0.6 and $k=$ 0.52; I) no case isolation with NB-based ${R}_t$ = 0.6 and $k=$ 0.18; J) perfect case isolation with NB-based ${R}_t$ = 0.6 and $k=$ 0.18; K) no case isolation with NB-based ${R}_t$ = 0.6 and $k=$ 0.03; L) perfect case isolation with NB-based ${R}_t$ = 0.6 and $k=$ 0.03. An onset-to-outcome delay of 0 days corresponds to counting days from the date of symptom onset of the last detected case. Red cells denote lower confidence that the outbreak is over following a 42-day period of no cases’ being detected after the outcome of the last detected case, while green cells denote higher confidence.

Our simulations showed that the underlying offspring distribution during the “decline” period of the outbreak was the most determinant factor in how long it took to reach 95% end-of-outbreak confidence. The waiting time was longer when the ${R}_t$ value was higher. On the other hand, a higher level of overdispersion (individual-level variation of transmission) in the offspring distribution led to shorter waiting times to reach 95% certainty. Outbreaks with no overdispersion tended to have a consistent duration and number of cases. However, outbreaks with high overdispersion were often shorter with a smaller final epidemic size; but by chance, they could also last longer with a considerable number of cases (Web Figure 2).

We found that the length of the onset-to-outcome delay phase affected the waiting time to reach 95% end-of-outbreak confidence, with longer onset-to-outcome delays leading to shorter waiting times. On the other hand, the influence of the reporting rate was elevated when there was a long onset-to-outcome delay, with higher reporting rates leading to shorter waiting times. However, the waiting times were constant when we used the symptom onset day as the start date of the waiting time to reach 95% certainty that an outbreak was over. For example, the waiting times to reach 95% end-of-outbreak confidence for Poisson-distribution–based outbreaks with ${R}_t$ = 0.6 for all tested values of reporting rate were around 107 days (no-case-isolation scenario) and 72 days (perfect-case-isolation scenario) (Figure 2A and Figure 2B, respectively).

The assumption about whether all detected and undetected cases or only the last detected case and subsequent undetected cases could still contribute to onward transmission or not (no case isolation or perfect case isolation) also substantially affected the waiting time. Assuming perfect isolation of past cases led to shorter waiting times, as expected. Finally, sensitivity analyses suggested that the developed framework was not robust to misspecification of the value of the reproduction number during the “decline” period (Web Figures 3 and 4).

We calculated the confidence that an EVD outbreak was over after 42 days following the outcome of the last detected case (Figure 3), which is the current WHO criterion for declaring the end of an EVD outbreak. Our simulations showed that in most scenarios considered, the current WHO criterion corresponded to end-of-outbreak confidence well below 95%. The corresponding confidence reached 95% only when the onset-to-outcome delay phase was very long (at least 2 or 3 weeks, which is unusual) and the reporting rate was high. However, when ${R}_t$ was low and all detected cases were entirely isolated (perfect case isolation), the end-of-outbreak confidence reached 95% in more parameter combinations. Once again, misspecification of the value of ${R}_t$ during the “decline” period strongly affected these results (Web Figures 5 and 6). An additional analysis accounting for the 90-day enhanced surveillance after the end-of-outbreak declaration for EVD (8) showed that the estimated end-of-outbreak confidence was more than 95% after 132 days (42 + 90 days), except when the value of ${R}_t$ was high (Web Figure 7).

## DISCUSSION

We developed a simulation-based framework with which to estimate the confidence that an outbreak is over after a certain amount of time with no new cases reported has passed. Our simulations showed that the reporting rate and the time between the symptom onset and the outcome of the last detected case are important factors that need to be considered in assessing the end of an outbreak. We applied this simulation-based framework to analyze a range of simulated EVD outbreaks with different levels of superspreading consistent with EVD epidemiology. We also explored different scenarios spanning extreme assumptions about the effectiveness of case isolation (from no case isolation to perfect case isolation).

Our results showed that under the current WHO criterion for declaring the end of an EVD outbreak, the confidence that an outbreak is over is still low (<46%) in most scenarios. Thus, a more robust end-of-outbreak criterion, supported by quantitative evidence, is needed to minimize the risk of flare-ups of cases after the end-of-outbreak declaration. The multiple flare-ups of cases that occurred after end-of-outbreak declarations at the tail end of the West African EVD epidemic also highlight this problem (35).

Some of the previous EVD outbreak flare-ups happened 51, 68, 78, and 80 days after the end-of-outbreak declarations (35). These flare-ups highlight the importance of the WHO recommendation of 90 days of enhanced surveillance after the 42 days of waiting time in declaring the end of an EVD outbreak. Our simulations also support the importance of this enhanced surveillance period. The estimated end-of-outbreak confidence after this 132-day (42 + 90 days) period from the outcome of the last detected case was higher than 95%, except in rare scenarios with a high reproduction number during the “decline” period (Web Figure 2).

Our simulations showed that the estimated end-of-outbreak confidence was very sensitive to the value of ${R}_t$, the instantaneous reproduction number at time *t*, during the “decline” period. Therefore, monitoring of ${R}_t$ during an outbreak is very important for defining the end of an outbreak accurately. Especially in the “decline” period, ensuring that ${R}_t$ is reduced well below 1 is critical for bringing the outbreak to an end. Current methods available for estimating ${R}_t$ during an outbreak may suffer from imprecision. This imprecision generally occurs when case numbers are low or if there is uncertainty in the serial interval distribution estimates (34). Our simulations showed that the framework we developed is sensitive to estimated ${R}_t$. These factors combined emphasize the need for continued assessment of ${R}_t$ throughout the outbreak, particularly as case numbers decrease. Consequently, it is hard to define a single criterion for the end of an outbreak, given how influential ${R}_t$ is.

We found that the time between the symptom onset and the death or recovery of the last detected case greatly impacted the waiting time to declare the end of an outbreak. A short delay between onset and outcome would lead to a longer time needed to reach 95% end-of-outbreak confidence. This delay period varies between cases, in particular, depending on the outcome (mean onset-to-recovery and onset-to-death were estimated as 14.4–15.3 days and 6.2–8.8 days, respectively (33)). Hence, our results do not support the current WHO single criterion for declaring the end of an outbreak, irrespective of the outcome of the last detected case.

Our study also showed that the reporting rate plays an important role in assessing the end of an outbreak. In line with results published by Thompson et al. (26), a low reporting rate would lead to lower confidence in declaring the end of an EVD outbreak on day 42 after the outcome of the last case, that is, using the current WHO criterion. Hence, for outbreaks with low reporting rates, a longer waiting time would be needed to declare the end of an outbreak. However, we found that the dependency on the reporting rate became negligible as the time between the symptom onset and the outcome of the last detected cases decreased. This dependency suggests that using the symptom onset day of the last detected case, rather than the outcome day, as the baseline of the waiting time should be considered. Using the symptom onset day as the baseline may be more robust to reporting rate variability in the outbreak context. It would also account for all of the possible outcome scenarios for the last detected case (2 consecutive negative tests or safe burial), including any delays in the testing or delays in burial (8).

We tested our simulation-based framework under various assumptions regarding overdispersion in offspring distributions caused by superspreading events. Our simulations showed that the waiting time decreases as overdispersion increases. We also explored scenarios in which there is perfect case isolation and no case isolation of all detected cases. We found that the current WHO criterion will perform best in the perfect-case-isolation scenario. However, given the difficulty of controlling and isolating cases during an outbreak, this perfect-case-isolation scenario should be considered a best-case scenario and the no-case-isolation scenario a worst-case scenario.

We propose a 2-step quantitative framework for assessing the end of an outbreak. First, estimate the key outbreak parameters: ${R}_t$, the reporting rate, and the serial interval distribution. These can be estimated from outbreak data using widely available and established methods, which increasingly account for sparse data (34, 36–38). Second, implement the method developed in this study to determine the day on which the estimated end-of-outbreak confidence is deemed acceptable (in this study, >95%), and the outbreak can be declared over. The developed framework is generic; thus, it could be implemented for outbreaks of other pathogens, primarily if they are airborne or directly transmitted.

We recommend a new set of criteria using the symptom onset day of the last detected case as the baseline of the waiting time used to declare the end of an EVD outbreak. The symptom onset day is usually captured better and is less affected by diagnostic waiting times than the outcome day. However, given the sensitivity of the simulation framework to the value of ${R}_t$, it is difficult to suggest a single criterion for an end-of-outbreak declaration irrespective of ${R}_t$. Therefore, we make 3 general recommendations:

1. A shift of counting down to the end of an outbreak from 42 days from the outcome day to 63 days from the symptom onset day of the last detected case leading to a preliminary end-of-outbreak declaration.
2. Emphasis on the importance of adequately resourcing the enhanced 90-day surveillance after the preliminary end-of-outbreak declaration to ensure that any flare-ups are quickly detected and controlled before the final end-of-outbreak declaration (63 + 90 days after the onset of the last detected case).
3. Regular estimation and reestimation of the reproduction number, particularly in the decline phase.

Finally, our simulation framework does not cover some aspects of EVD transmission, which deserve to be highlighted. The framework developed did not consider additional cases that arise from less common transmission routes, such as migration, sexual transmission, and immunocompromised pregnant women (21). These caveats make the current policy of keeping active case detection up to 90 days after the end-of-outbreak declaration essential if the framework and model were to be adopted. Although the framework accounts for superspreading (by allowing overdispersion in the offspring distribution), we did not explicitly model superspreading events in the context of unsafe burial practices that lead to large-scale funeral exposures (31, 39). Nevertheless, the work presented here demonstrates the value of developing a quantitative framework to support objective assessments of the risk of flare-ups of cases after the end-of-outbreak declaration. It also highlights the limitations of the current WHO criterion for declaring the end of an outbreak of EVD.

## Supplementary Material

Web_Material_kwaa212
