# Supplementary material for: A Quantitative Framework for Defining the End of an Infectious Disease Outbreak: Application to Ebola Virus Disease
Source: Am J Epidemiol. 2020 Oct 14;190(4):642–51. doi: 10.1093/aje/kwaa212 (PMC8024054; doi:10.1093/aje/kwaa212)
Supplement: Web_Material_kwaa212 [file web_material_kwaa212.pdf]

Web Appendix

A Quantitative Framework for Defining the End of an Infectious Disease Outbreak: Application to Ebola Virus Disease

Bimandra A. Djaafara, Natsuko Imai, Esther Hamblion, Benido Impouma, Christl A. Donnelly, and Anne Cori

Contents

List of Tables 1

List of Figures 1

The end-of-outbreak declaration phase trajectories simulation process 2

Complete simulation scenarios 4

Summary of simulated outbreak data 6

Robustness of the framework to misspecification of instantaneous reproduction number value at the 'decline' period 8

Estimated end-of-outbreak confidence considering the WHO policy of 90 days of enhanced surveillance 13

References 16

List of Tables

Web Table 1 5

List of Figures

Web Figure 1 3

Web Figure 2 7

Web Figure 3 9

Web Figure 4 10

Web Figure 5 11

Web Figure 6 12

Web Figure 7 14

The end-of-outbreak declaration phase trajectories simulation process

Each end-of-outbreak declaration phase trajectory was simulated using the *project* function from the *projections* R package (1). The combination of the cases in the simulated outbreak trajectory and simulated undetected cases during the onset-to-outcome delay phase were used as the initial values of each simulation. The forward simulation process of the end-of-outbreak declaration phase trajectories is shown in **Web Figure 1** (next page). A total of 100,000 end-of-outbreak declaration phase trajectories were generated for each simulation scenario.

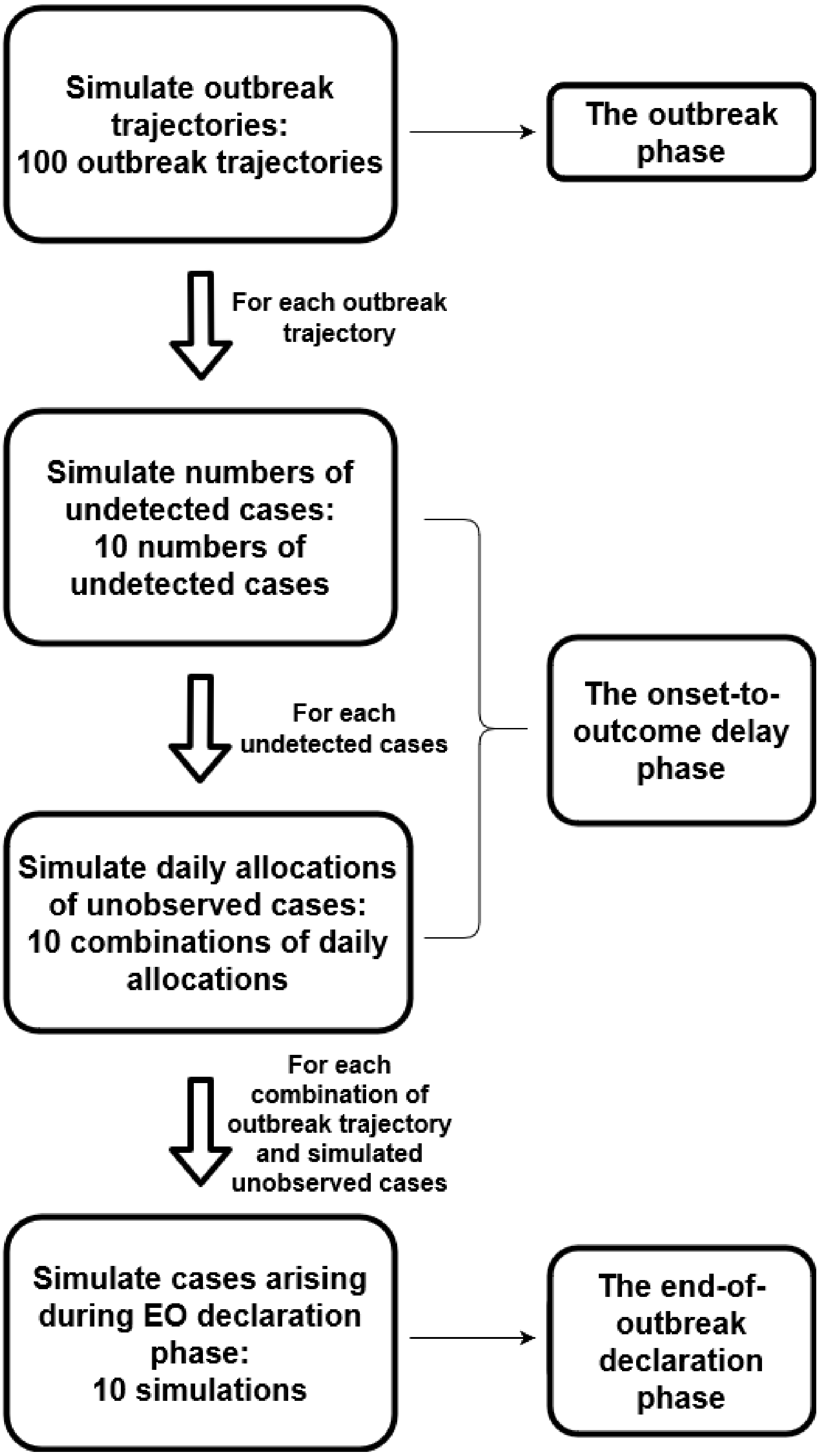

Web Figure 1. Forward simulation process for the end-of-outbreak declaration phase trajectories. Right panels denote phases where each simulation procedure was conducted.

Complete simulation scenarios

Complete combinations of all simulation scenarios of this study are shown in **Web Table 1** (next page).

Web Table 1. Complete combinations of all simulation scenarios – combination of simulated outbreak datasets used and end-of-outbreak framework simulations generated based on the simulated outbreak data are summarized.

Simulated Outbreaks			End-of-Outbreak Framework Simulations				Results Shown In:
**Offspring Distribution**	**Simulated Outbreak 'Decline' Period R_t**	**Overdispersion Parameter (If Negative Binomial)**	**End-of-Outbreak Simulation Framework R_t**	**Transmission Assumption**	**Reporting Rate**	**Length of Onset-to-Outcome Delay Phase (Days)**	
Poisson	0.6	-	0.6	No-case-isolation, Perfect-case-isolation	50%, 60%, 70%, 80%, 90%, 100%	0 (onset day as baseline), 1, 7, 14, 21	Figures 2 & 3
Poisson	0.3	-	0.3	No-case-isolation, Perfect-case-isolation	50%, 60%, 70%, 80%, 90%, 100%	0 (onset day as baseline), 1, 7, 14, 21	Figures 2 & 3
Poisson	0.9	-	0.9	No-case-isolation, Perfect-case-isolation	50%, 60%, 70%, 80%, 90%, 100%	0 (onset day as baseline), 1, 7, 14, 21	Figures 2 & 3
Negative binomial	0.6	0.03	0.6	No-case-isolation, Perfect-case-isolation	50%, 60%, 70%, 80%, 90%, 100%	0 (onset day as baseline), 1, 7, 14, 21	Figures 2 & 3
Negative binomial	0.6	0.18	0.6	No-case-isolation, Perfect-case-isolation	50%, 60%, 70%, 80%, 90%, 100%	0 (onset day as baseline), 1, 7, 14, 21	Figures 2 & 3
Negative binomial	0.6	0.52	0.6	No-case-isolation, Perfect-case-isolation	50%, 60%, 70%, 80%, 90%, 100%	0 (onset day as baseline), 1, 7, 14, 21	Figures 2 & 3
Poisson	0.6	-	0.3	No-case-isolation, Perfect-case-isolation	50%, 60%, 70%, 80%, 90%, 100%	0 (onset day as baseline), 1, 7, 14, 21	Web Figures 3 & 5
Poisson	0.6	-	0.9	No-case-isolation, Perfect-case-isolation	50%, 60%, 70%, 80%, 90%, 100%	0 (onset day as baseline), 1, 7, 14, 21	Web Figures 3 & 5
Negative binomial	0.6	0.03	0.3	No-case-isolation, Perfect-case-isolation	50%, 60%, 70%, 80%, 90%, 100%	0 (onset day as baseline), 1, 7, 14, 21	Web Figures 4 & 6
Negative binomial	0.6	0.03	0.9	No-case-isolation, Perfect-case-isolation	50%, 60%, 70%, 80%, 90%, 100%	0 (onset day as baseline), 1, 7, 14, 21	Web Figures 4 & 6

Summary of simulated outbreak data

Using the algorithm described in the main text (Methods), we simulated Ebola virus disease (EVD) outbreak data with various outbreak offspring distribution assumptions. We simulated EVD outbreak offspring distribution with no overdispersion (Poisson), low overdispersion (negative binomial $k = 0.52$ (2)), medium overdispersion (negative binomial $k = 0.18$ (3)) and high overdispersion (negative binomial $k = 0.03$ (2)) with $R_t = 0.6$ in the 'decline' period of the outbreak. For each outbreak scenario, 1,000 outbreak trajectories were simulated using the *project* function from the *projections* package of R programming language (1). The summary of the simulated outbreak data for each scenario: length of the outbreak and the number of cases generated during the outbreak is presented in **Web Figure 2** (next page). Outbreaks with no overdispersion had a median of 605 total cases and lasted a median of 237 days. Outbreaks with high overdispersion led to a skewed distribution of the number of cases generated and the length of the outbreak. The median for each respective variable was 23 cases and 49.5 days. However, by chance, outbreaks with high overdispersion can generate up to almost 20,000 cases that lasts up to 308 days.

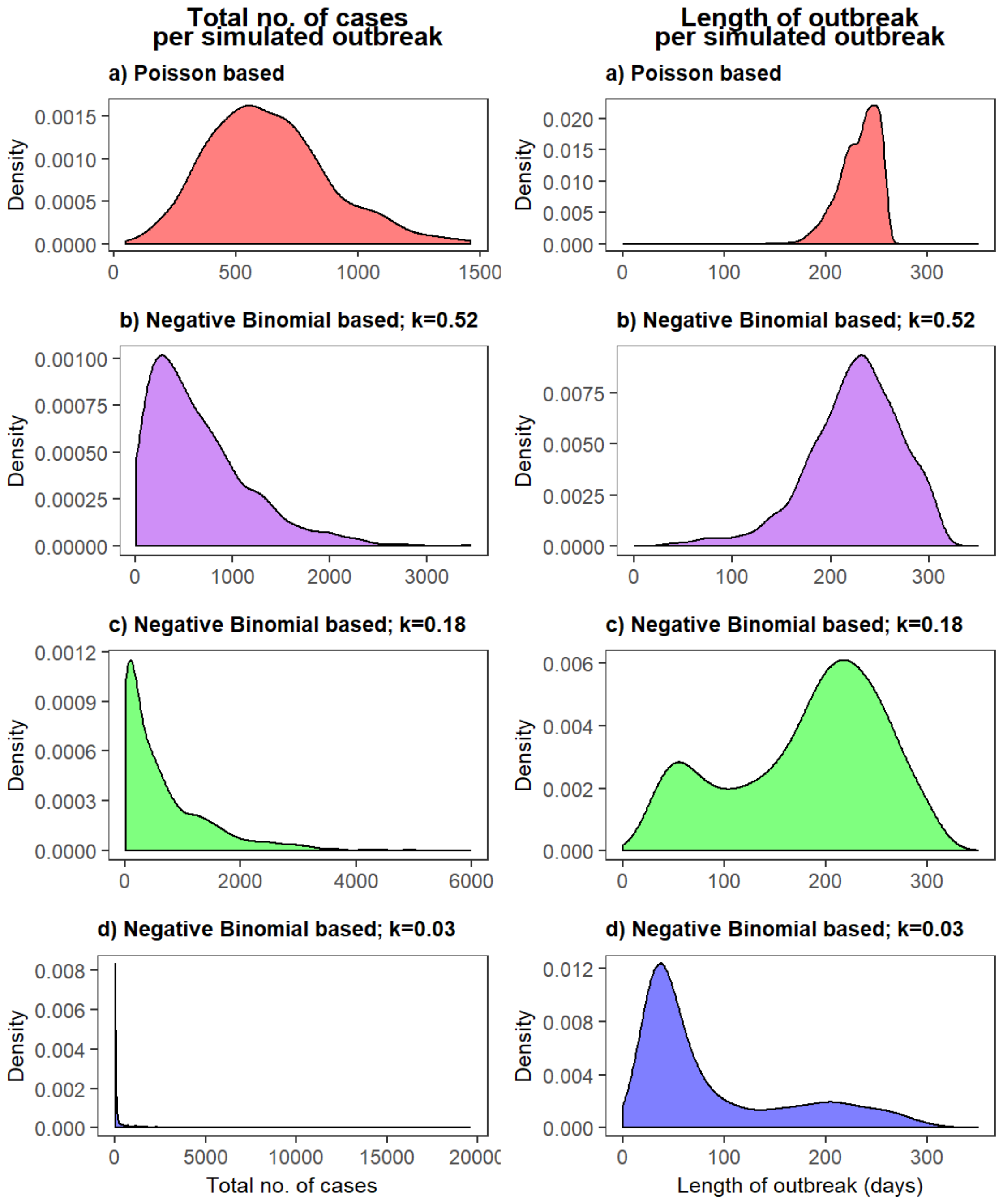

Web Figure 2. Summary of simulated outbreak data based on Poisson offspring distribution and negative binomial offspring distribution with low, medium, and high overdispersion. Graphs on the left side represent the distribution of the total number of cases of the simulated outbreak data for each outbreak offspring distribution (note the different axis limits). Graphs on the right side represent the length of the simulated outbreak data for each outbreak offspring distribution.

Robustness of the framework to misspecification of instantaneous reproduction number value at the ‘decline’ period

The current tools to estimate the instantaneous reproduction number can be sensitive to the value of reporting rate, the serial interval distribution and the size of the sliding window for the estimation period (4). We therefore tested the robustness of our quantitative framework on the uncertainty of the instantaneous reproduction number estimates in the ‘decline’ period. Sensitivity analyses were conducted by simulating the ‘decline’ phase of the epidemic with a $R_t = 0.6$, but under- ($R_t = 0.3$) or overestimating ($R_t = 0.9$) this true value in the same period in the implementation of the simulation framework. Sensitivity analyses on the waiting time to reach 95% end-of-outbreak confidence for outbreaks with no overdispersion and high overdispersion are presented in **Web Figures 3 & 4**. Sensitivity analyses results of the end-of-outbreak confidence after 42 days, following the onset/outcome of the last detected case are presented in **Web Figures 5 & 6**.

Our simulations show that the end-of-outbreak confidence is sensitive to the value of the instantaneous reproduction number used for the simulations. Longer waiting times to end-of-outbreak declaration were expected based on simulation results assuming a higher R_t. The waiting times are mostly exceeded 250 days when R_t was overestimated and no overdispersion were assumed. On the other hand, waiting times to end-of-outbreak declaration when assuming R_t was underestimated were shorter based on simulation results.

When R_t was overestimated, we would expect a lower end-of-outbreak confident following 42 days after the onset/outcome of the last detected case. Conversely, a higher confidence is expected when R_t was underestimated.

The sensitivity of our estimates to the reporting rate when the length of the onset-to-outcome delay phase is relatively long is still observed. However, in the outbreak with high overdispersion (**Web Figures 4 and 6**), the estimates are more robust to the reporting rate compared to the outbreak with low overdispersion. When the length of the onset-to-outcome delay phase is relatively long, the waiting time to end-of-outbreak declaration is shorter and the end-of-outbreak confidence after day 42 following the outcome of the last case decreases as the reporting rate increases. Using the symptom onset day as the baseline to end-of-outbreak declaration gave robust estimates of waiting times and probabilities of cases arising in the future.

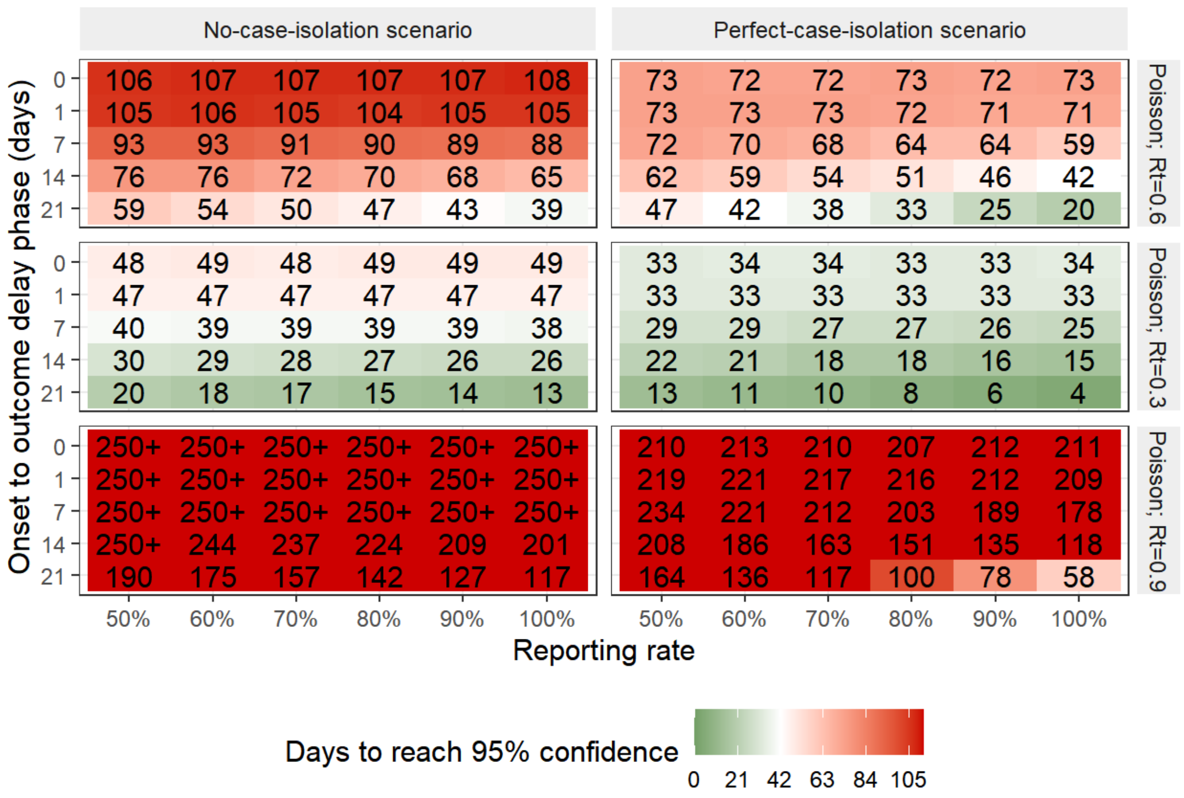

Web Figure 3. Sensitivity analysis of our framework on the value of the instantaneous reproduction number using simulated outbreaks with Poisson offspring distribution ($R_t = 0.6$) representing an outbreak with no overdispersion. The value inside each cell denotes the number of days (from the outcome of the last reported case) until the end-of-outbreak confidence reaches 95%. An onset-to-outcome delay of zero days corresponds to counting days from the symptom onset day of the last detected case. From the top row to the bottom row: 1) Framework simulations with ‘decline’ period $R_t = 0.6$ – same as simulated outbreak and no misspecification of R_t in the decline phase; 2) end-of-outbreak assessments with assumed ‘decline’ period $R_t = 0.3$ – underestimation of simulated outbreak R_t; and 3) end-of-outbreak assessments with assumed ‘decline’ period $R_t = 0.9$ – overestimation of simulated outbreak R_t.

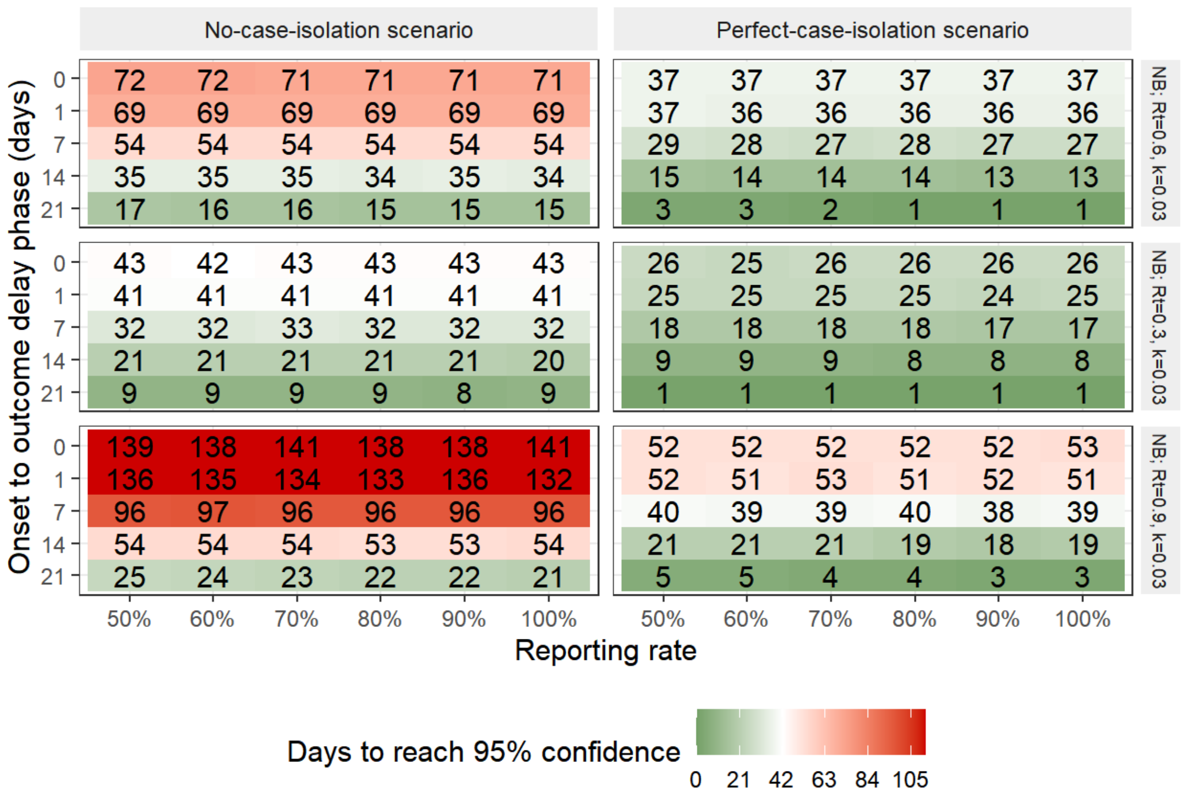

Web Figure 4. Sensitivity analysis of our framework to the value of the instantaneous reproduction number using simulated outbreaks with negative binomial offspring distribution ($R_t = 0.6,\ k = 0.03$) representing outbreaks with high overdispersion. The value inside each cell denotes the number of days (from the outcome of the last reported case) until the end-of-outbreak confidence reaches 95%. An onset-to-outcome delay of zero days corresponds to counting days from the symptom onset day of the last detected case. From the top row to the bottom row: 1) Framework simulations with 'decline' period $R_t = 0.6$ – same as the simulated outbreak and no misspecification of R_t in the decline phase; 2) end-of-outbreak assessments with assumed 'decline' period $R_t = 0.3$ – underestimation of simulated outbreak R_t; and 3) end-of-outbreak assessments with assumed 'decline' period $R_t = 0.9$ – overestimation of simulated outbreak R_t.

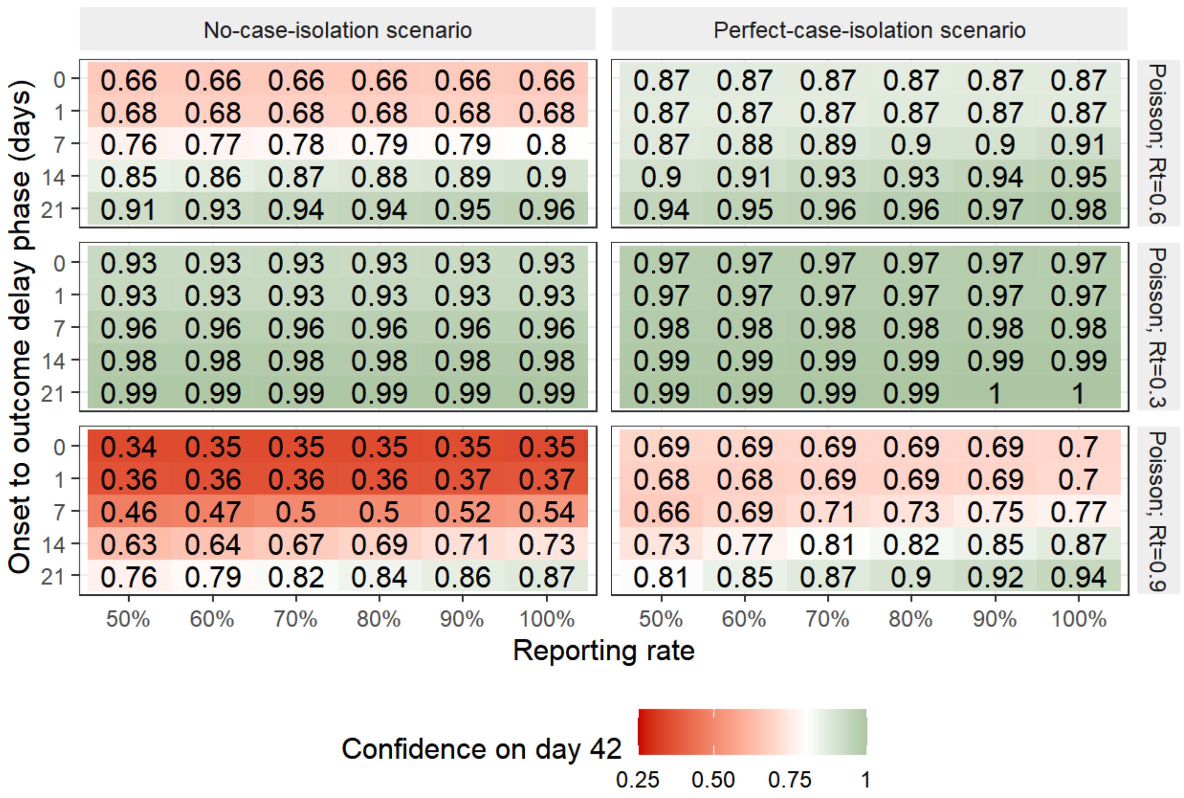

Web Figure 5. Sensitivity analysis of our developed framework to the value of instantaneous reproduction number using simulated outbreaks with Poisson offspring distribution ($R_t = 0.6$) representing outbreak with no overdispersion. The value inside each cell denotes the confidence that the outbreak is over following 42-day period of no cases detected after outcome of the last detected case. An onset-to-outcome delay of zero days corresponds to counting days from the symptom onset day of the last detected case. From the top row to the bottom row: 1) Framework simulations with 'decline' period $R_t = 0.6$ – same as simulated outbreak and no misspecification of R_t in the decline phase; 2) end-of-outbreak assessments with assumed 'decline' period $R_t = 0.3$ – underestimation of simulated outbreak R_t; and 3) end-of-outbreak assessments with assumed 'decline' period $R_t = 0.9$ – overestimation of simulated outbreak R_t.

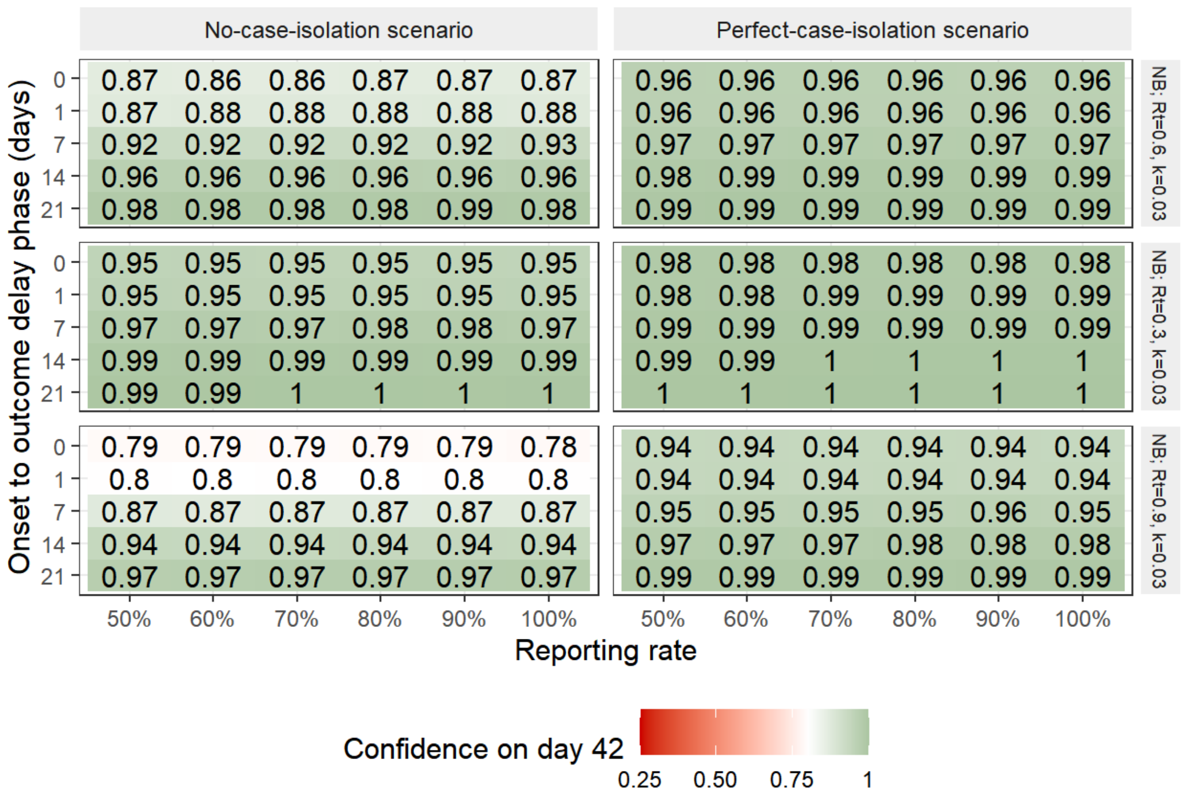

Web Figure 6. Sensitivity analysis of our developed framework to the value of instantaneous reproduction number using simulated outbreaks with negative binomial offspring distribution ($R_t = 0.6,\ k = 0.03$) representing outbreaks with high overdispersion. The value inside each cell denotes confidence that the outbreak is over following 42-day period of no cases detected after the outcome of the last detected case. An onset-to-outcome delay of zero days corresponds to counting days from the symptom onset day of the last detected case. From the top row to the bottom row: 1) Framework simulations with 'decline' period $R_t = 0.6$ – same as the simulated outbreak and no misspecification of R_t in the decline phase; 2) end-of-outbreak assessments with assumed 'decline' period $R_t = 0.3$ – underestimation of simulated outbreak R_t; and 3) end-of-outbreak assessments with assumed 'decline' period $R_t = 0.9$ – overestimation of simulated outbreak R_t.

Estimated end-of-outbreak confidence considering the WHO policy of 90 days of enhanced surveillance

WHO policy for the end-of-outbreak of EVD includes additional 90 days of enhanced surveillance after the end-of-outbreak declaration. We calculated the confidence that the outbreak is over after 90 days of enhanced surveillance (day 42 + 90 after the onset/outcome of the last case) for every simulation scenario (**Web Figure 7**). The estimated end-of-outbreak confidence after the additional 90 days of enhanced surveillance reached 95% in most of the simulation scenarios except in the third scenario, when the R_t of the Poisson distribution was 0.9.

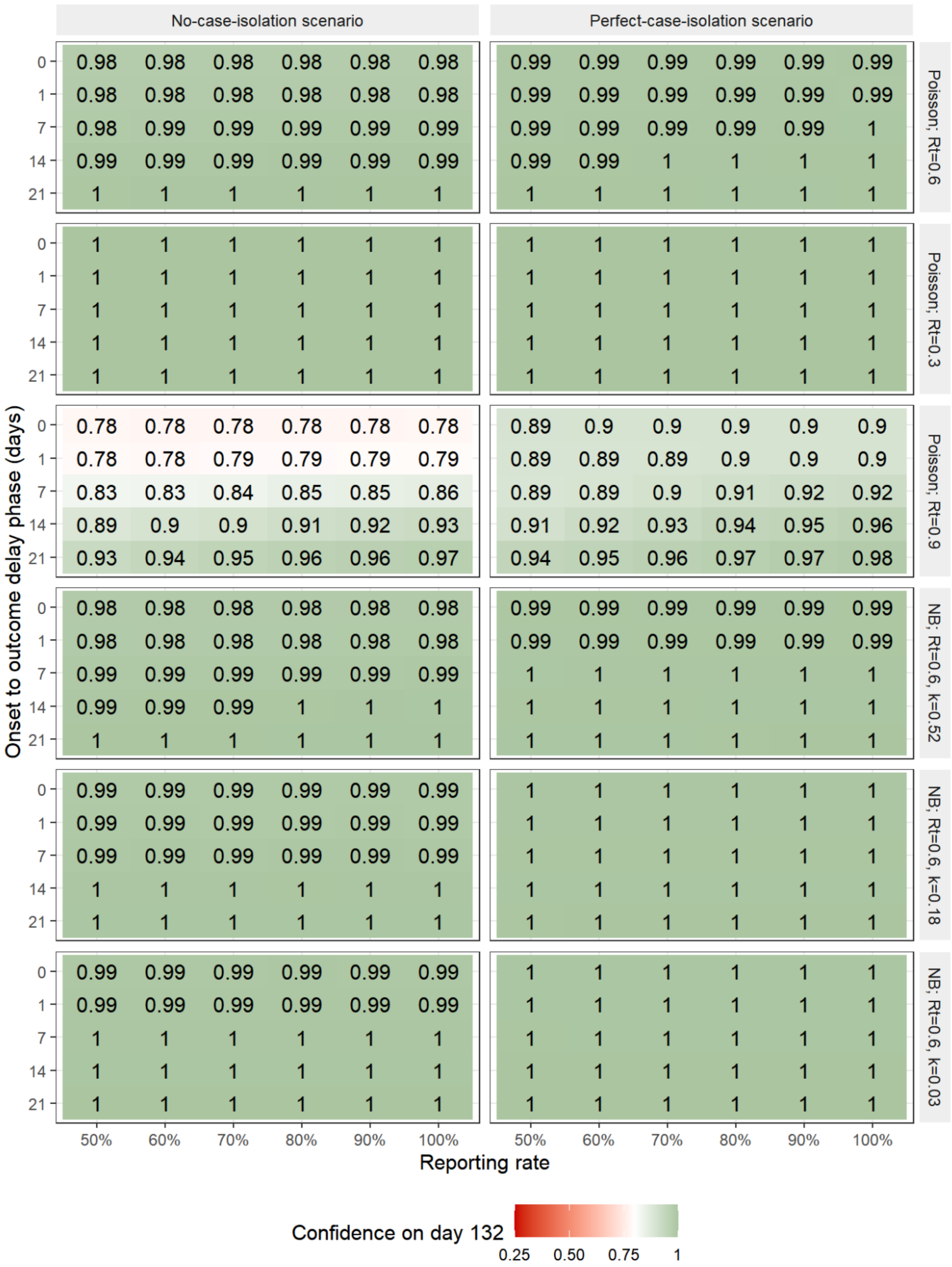

Web Figure 7. Confidence that the outbreak is over following 132 (42 + 90) days period after the outcome of the last detected case for various offspring distributions during the ‘decline’ period (from the top row to the bottom row: 1) Poisson-based, $R_t =$

0.6; 2) Poisson-based, $R_t = 0.3$; 3) Poisson-based, $R_t = 0.9$; 4) negative binomial-based, $R_t = 0.6$ and $k = 0.52$; 5) negative binomial-based, $R_t = 0.6$ and $k = 0.18$; and 6) negative binomial-based, $R_t = 0.6$ and $k = 0.03$) and for the no-case-isolation (left) and perfect-case-isolation (right) scenarios, as a function of the length of the reporting rate and the onset-to-outcome delay phase. An onset-to-outcome delay of zero days corresponds to counting days from the onset day of the last detected case. Red cells denote lower confidence that the outbreak is over following 132 days period after the onset/outcome of the last detected case while green cells denote higher confidence.

References

1. Jombart T, Nouvellet P. projections: Project Future Case Incidence (R package version 0.3.1). 2018. https://cran.r-project.org/package=projections.
2. International Ebola Response Team, Agua-Agum J, Ariyarajah A, et al. Exposure Patterns Driving Ebola Transmission in West Africa: A Retrospective Observational Study. *PLOS Med*. 2016;13(11):e1002170.
3. Althaus CL. Ebola superspreading. *Lancet. Infect. Dis.* 2015;15(5):507–8.
4. Cori A, Ferguson NM, Fraser C, et al. A new framework and software to estimate time-varying reproduction numbers during epidemics. *Am J Epidemiol.* 2013; 178(9):1505–12.
